# Alterations in the Corneal Nerve and Stem/Progenitor Cells in Diabetes: Preventive Effects of Insulin-Like Growth Factor-1 Treatment

**DOI:** 10.1155/2014/312401

**Published:** 2014-02-18

**Authors:** Hiroki Ueno, Takaaki Hattori, Yuta Kumagai, Noboru Suzuki, Satoki Ueno, Hitoshi Takagi

**Affiliations:** ^1^Department of Ophthalmology, St. Marianna University School of Medicine, 2-16-1 Sugao, Miyamae-ku, Kawasaki, Kanagawa 216-8511, Japan; ^2^Department of Ophthalmology, Tokyo Medical School of Medicine, 6-7-1 Nishishinjuku, Shinjuku-ku, Tokyo 160–0023, Japan; ^3^Departments of Immunology and Medicine, St. Marianna University School of Medicine, 2-16-1 Sugao, Miyamae-ku, Kawasaki, Kanagawa 216-8511, Japan

## Abstract

This study aimed to investigate whether corneal nerve and corneal stem/progenitor cells are altered in insulin-like growth factor-I (IGF-I-) treated individuals with diabetes. A group consisting of db/db mice with type 2 diabetes mellitus (DM) and a wild-type group were assessed by neural and corneal stem/progenitor cell markers immunostaining and real-time PCR. Moreover, the expression of corneal nerve and stem/progenitor cell markers was examined in IGF-1-treated diabetic mice. Compared with a normal cornea, swelling and stratification of the corneal epithelium were noted in db/db mice. Beta-III tubulin immunostaining revealed that the corneal subbasal plexuses in diabetic mice were thinner with fewer branches. mRNA expression levels of *Hes1*, *Keratin15*, and *p75* (corneal stem/progenitor cell markers) and the intensity and number of positive cells of Hes1 and Keratin19 immunostaining diminished in the diabetic corneas. Compared with the subbasal nerve density in the normal group, a decrease in the diabetic group was observed, whereas the corneal subbasal nerve density increased in IGF-1-treated diabetic group. The decreased expression of Hes1 and Keratin19 was prevented in IGF-1-treated diabetic group. Our data suggest that corneal nerve and stem/progenitor cells are altered in type 2 DM, and IGF-I treatment is capable of protecting against corneal damage in diabetes.

## 1. Introduction

Corneolimbal epithelial progenitor/stem cells are a small subpopulation of oligopotent cells that are primarily located in the basal epithelial layer of the limbus. They produce undifferentiated progeny with limited proliferative potential that centripetally migrate from the periphery of the cornea to replace cells that desquamate during normal life [1–7]. Corneolimbal stem cells primarily reside in the palisades of Vogt in a niche that maintains their stemness by producing a unique anatomical and functional milieu [8, 9]. Although in humans the exact anatomical location of the niche is considered to be in the limbus, it has recently been proposed that epithelial progenitor/stem cells of equal potency are distributed throughout the entire ocular surface in other mammals [10]. Detection of corneal epithelial stem cells is the object of controversy among several groups because corneal progenitor/stem cell markers remain undetermined. Hes1 is known to be important for maintaining the self-renewal ability of progenitors and repressing the commitment of multipotent progenitor cells to a neuronal fate. In addition, Nakamura et al. reported that Hes1 regulates the corneal development and homeostatic function of corneal stem/progenitor cells [11]. p75NTR is a member of the tumor necrosis factor receptor superfamily, originally identified as a receptor for neurotrophins, and is expressed in the nervous system. p75 is also selectively expressed by basal epithelial cells in the corneal limbus; however, it is absent in the central cornea. ATP-binding cassette subfamily G member 2 (ABCG2) and keratin 19 and 15 as putative corneal epithelial stem/progenitor cell-associated molecules are also expressed in the corneal limbal basal cells.

Diabetes mellitus is a systemic disease that is characterized by chronic hyperglycemia [12, 13]. Ocular complications, such as retinopathy, cataract, and glaucoma, are well known. In particular, patients with diabetic keratopathy (DK) show various symptoms such as persistent corneal epithelial defects [14–17]. Alterations in the epithelial basement membrane, insufficient tear secretion, and neuropathy-associated denervation have been linked to abnormalities of the ocular surface.

Diabetes/diabetes (db/db) mice are generally used to study the pathophysiology of type 2 diabetes. These mice have mutations in the genes that code for the leptin receptor, which is primarily localized in the hypothalamic region where it regulates the body weight and energy balance [18]. Db/db mice are hyperglycemic, hyperinsulinemic, and insulin resistant, and they demonstrate impaired tissue inflammatory responses in the cornea [19]. These mice have sensory nerve conduction deficits, small sensory nerve fiber neuropathy, and intraepidermal sensory nerve fiber loss [20]. Several studies have shown that skin wound healing is impaired in db/db mice, which is associated with a dysregulated inflammatory response [21]. To the best of our knowledge, there have been only a few reports that have focused on the influence of corneal progenitor/stem cells in neurodegenerative disorder such as type 2 diabetes. We have previously reported that corneolimbal stem cells significantly reduce after depletion of sensory nerves by electrocoagulation of the ophthalmic branch of the trigeminal nerve [22, 23]. Our data suggested a critical role of innervation in maintaining stem cells and/or the stem cell niche. The purpose of this study was to elucidate whether putative corneal stem/progenitor cells are altered in type 2 diabetic corneas and understand the pathogenesis of DK.

The prevention and amelioration of various complications caused by type 2 diabetes have been investigated. The insulin-like growth factor (IGF) system has been shown to play a role in diabetic retinopathy [24]. In previous studies, recombinant human insulin-like growth factor-I (rhIGF-I) has been shown to enhance the proliferation of several cell lines and protect cells from apoptosis induced by various factors [24–30]. We further aimed to assess and clarify whether IGF-I treatment is capable of protecting against corneal nerve damage and corneal stem/progenitor cells in diabetes. Our data demonstrate that sensory nerve degeneration of cornea affects stem cell homeostasis and leads to a significant increase in the number of corneolimbal stem/progenitor cells and corneal nerve in the rhIGF-1-treated diabetes. Herein, we hypothesized that there is a therapeutic potential of IGF-1 administration in neurodegenerative diseases such as type 2 diabetes.

## 2. Methods

### 2.1. Animals

Twelve-week-old male leptin receptor mutant (db/db) mice on a C57Bl/6 background and 12-week-old male C57BL/6 mice were obtained from the Jackson Laboratory (Bar Harbor, ME, USA). Db/db mice are widely used as models of type 2 diabetes. The research protocol conformed to the standards of the Association for Research in Vision and the Ophthalmology Statement for the Use of Animals in Ophthalmic and Vision Research and was approved by the Animal Care Facility of St. Marianna University School of Medicine.

### 2.2. Retrobulbar Administration of rhIGF-I

rhIGF-I was purchased from PeproTech (Rocky Hill, NJ, USA). rhIGF-I was reconstituted in phosphate-buffered saline (PBS) containing 0.1% bovine serum albumin (BSA) and stored at −80°C until use according to the manufacturer's instructions. In brief, the mice were anesthetized and placed in a stereotaxic frame (Narishige Scientific Instrument Lab, Tokyo, Japan). For rhIGF-1 treatment of the diabetic group, 11-week-old ob/ob mice were administered a retrobulbar injection of 100 *μ*g/kg rhIGF-I twice daily for 7 days. On day 7 of the postexperimental procedure, the corneas from diabetic animals (*n* = 8) were enucleated.

### 2.3. Immunohistochemistry

Cryostat sections of 5 *μ*m thickness were prepared from tissue that was embedded in Tissue-Tek OCT compound (Sakura Finetek USA, Inc., Torrance, CA, USA). After fixation with cold acetone for 10 min, the tissues were incubated with 10% goat serum containing 0.1% Triton X-100 and 1% BSA (room temperature (RT); 1 h) for Cytokeratin 19 (CK19) and with 1% BSA and goat serum (RT; 30 min) for hairy enhancer of split 1 (Hes1) [11]. For the CK19 staining, overnight incubation with a mouse monoclonal antibody against CK19 (1 : 200) (Abcam plc, Cambridge, MA, USA) was performed. For Hes1 staining, a rabbit anti-Hes1 polyclonal antibody (1 : 1,000) was used (from Dr. T. Sudo, Toray Industries, Inc., Tokyo, Japan). These sections were subsequently incubated (RT, 1 h) with the appropriate primary antibody and washed 3 times in PBS containing 0.15% Triton X-100 for 15 min. Antibody binding was detected by Alexa 488 or Alexa 594 conjugated secondary antibodies (Life Technologies GmbH, Darmstadt, Germany). The slides were washed 3 times in PBS containing 0.15% Triton X-100 for 15 min, coverslipped with antifading mounting medium containing 4,6-diamidino-2-phenylindole (Vectashield, Vector Laboratories, Inc., Burlingame, CA, USA), and examined under a confocal microscope (LSM-510, Carl Zeiss Microscopy GmbH, Jena, Germany). For whole-mount immunofluorescence corneal staining, freshly excised corneas including the limbus were harvested and fixed in 4% paraformaldehyde, stained with anti-*β* tubulin-III antibody (1 : 200) (Rabbit anti-*β* tubulin-III polyclonal antibody, EMD Millipore Corporation, Billerica, MA, USA), and incubated with a secondary antibody that was conjugated with fluorescein isothiocyanate (1 : 200) (Donkey anti-rabbit IgG, Santa Cruz Biotechnology, Inc., Santa Cruz, CA, USA). Radial cuts were prepared in the cornea so that it could be flattened by a coverslip, and the cornea was mounted using the mounting medium (Vectashield, Vector Laboratories, Inc., Burlingame, CA, USA). Images were taken at 400× magnification. Control incubations were performed with the appropriate normal mouse or rabbit IgGs at the same concentration as the primary antibody.

### 2.4. Real-Time Polymerase Chain Reaction (PCR)

RNA was isolated (RNeasy Micro Kit; QIAGEN Inc., Valencia, CA, USA) and reverse-transcribed (Superscript III Kit; Life Technologies Corporation). Real-time PCR was performed with a PCR mix (TaqMan Universal PCR Master Mix; Life Technologies Corporation, Carlsbad, CA) and preformulated primers for *Abcg2* (assay ID Mm00496364_m1), *Hes1* (assay ID Mm00468601_m1), *Keratin15* (assay ID Mm00492972_m1), *p75* (assay ID Mm01309635_m1), and *18S ribosomal RNA* (assay ID Mm03928990_g1) (all Applied Biosystems, Austin, TX, USA). The* 18S ribosomal *RNA was used as the endogenous reference for each reaction. The results were derived from the relative threshold cycle (Ct) by the deltadelta Ct method and normalized by *18S ribosomal RNA* used as an internal control. The relative messenger RNA (mRNA) level in the normal corneas (normal group) was used as the normalized control for the groups with diabetes.

### 2.5. Quantitative Analysis

Image J 1.45 (provided program from National Institutes of Health) was used to create stacked images and calculate the corneal nerve density for the subbasal nerve branches of normal, diabetic, and rhIGF-1-treated mice. All subbasal nerve branches of the stacked corneal images were traced. Corneal nerve fiber density was subsequently determined with the length of the nerve fibers in each 1 mm^2^ area. The differences in corneal nerve fiber density between the two consecutive groups of mice were compared by the analysis of variance using a statistical analysis software package (SSRI Co., Ltd., Tokyo, Japan).

### 2.6. Statistical Analysis

Statistical evaluation of data was performed with the unpaired *t*-test and the nonparametric Mann-Whitney *U* test for comparisons among groups. The data are presented as mean ± standard error of the mean and the value of *P* < 0.05 was considered statistically significant.

## 3. Results

### 3.1. Alterations in Ocular Properties

**Figure 1:**
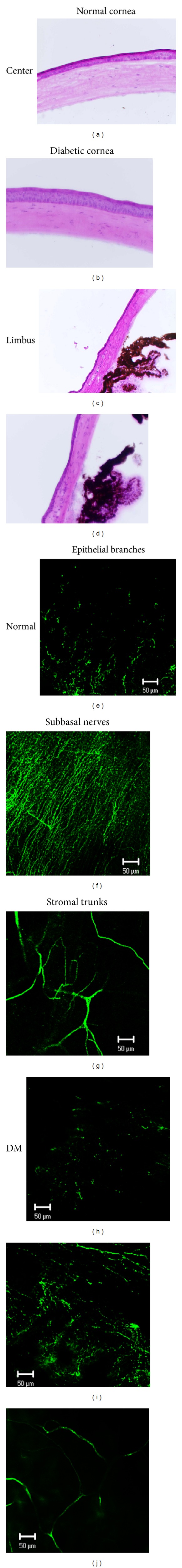
Ocular properties in ob/ob mice. Representative histological photomicrographs of hematoxylin and eosin-stained globe sections that were obtained from normal and 12-week-old diabetic mice. Swelling and stratification of the diabetic corneal epithelium are noted in comparison with the normal cornea. Magnification: 200x. Representative biomicroscopy of a normal cornea ((a) and (c)) and of a diabetic mouse cornea after 12 weeks ((b) and (d)). Representative histological photomicrographs of corneal nerves that were immunostained with an anti-*β*-III-tubulin fluorescein isothiocyanate-conjugated antibody showing the epithelial nerve (left column), subbasal nerve plexus (middle column), and stromal nerves (right column) in the normal eye ((e), (f), and (g)) and in a 12-week-old diabetic mouse eye ((h), (i), and (j)). Note the development of marked nerve degeneration after 12 weeks when the nerves appear faint and interrupted. Magnification: 200x.

The morphology of the corneal epithelium and stroma was compared between a normal cornea (Figure 1(a): the center and Figure 1(c): the limbus) and the diabetic cornea of 12-week-old db/db mice (Figure 1(b): the center and Figure 1(d): the limbus) with hematoxylin and eosin staining, and the swelling and stratification of the diabetic corneal epithelium, both in the center (Figure 1(b)) and limbus (Figure 1(d)), were noted compared with that of the normal corneas (Figures 1(a) and 1(c)). In the diabetic group, the subbasal nerve plexus did not exhibit its characteristic branching pattern. The corneal subbasal nerve branches in the diabetic group (Figure 1(i)) appeared faint and disrupted. Moreover, no clear nerve structure of the epithelial branches could be significantly detected in the diabetic group (Figure 1(h)) compared with that in the normal group (Figure 1(e)).

### 3.2. mRNA Expression and Immunostaining of Stem Cell Markers

**Figure 2:**
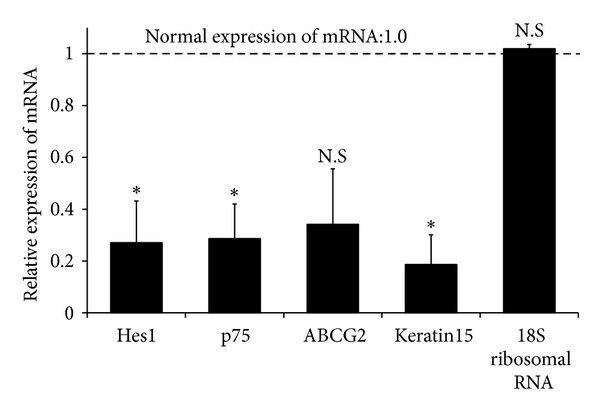
Diminished mRNA expression levels of stem cell markers in diabetic cornea. The transcript levels of *p75*, hairy enhancer of split 1 (*Hes1*), *Abcg2*, and *Keratin15* in the cornea are shown. The mRNA expression levels of *p75, Hes1*, and *Keratin15* significantly decreased in diabetic corneas. A reduction of *Abcg2* was also observed, although this was not statistically significant. *18S ribosomal RNA* expression showed no significant difference between diabetic corneas and normal corneas. Statistical significance for mRNA levels is calculated on fold-change values over those of diabetic corneas. The dotted line represents the mRNA expression levels in normal corneas (normal expression of mRNA: 1.0). Statistical evaluation of data was performed with the unpaired *t*-test. The bars represent the mean ± standard error of the mean (SEM) (*P < 0.05).

**Figure 3:**
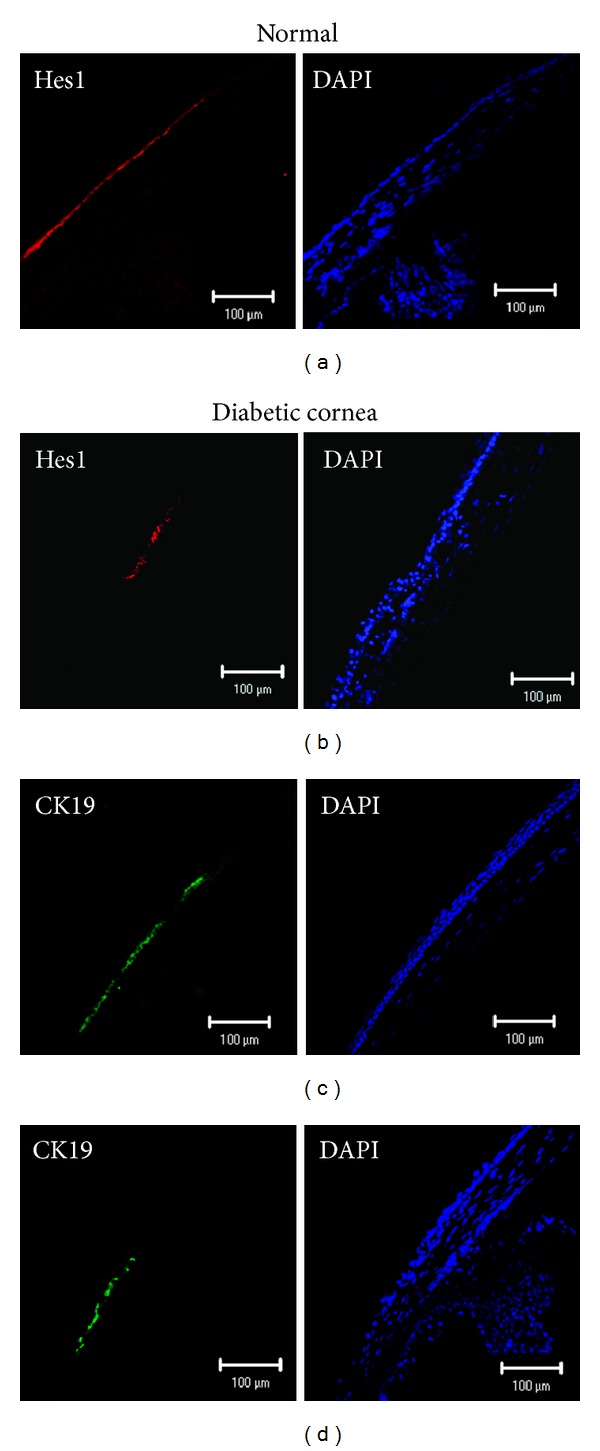
Decreased expression levels of corneal progenitor/stem cell markers in diabetes. Representative immunostained micrographs of the cross-sections of the normal ((a) and (c)) and diabetic ((b) and (d)) corneas showing the expression of stem cell markers. Expression levels of Hes1 (b) and Keratin19 (d) decreased in diabetic eyes after 12 weeks compared with those in the normal eyes ((a) and (c)).

We analyzed the expression levels of *Abcg2, Hes1, p75,* and* Keratin15* in normal and diabetic corneas using real-time PCR (Figure 2). Decreased mRNA expression levels of *p75* (3-fold; *P* = 0.019), *Hes1* (3-fold: *P* = 0.035), and *Keratin15* (5-fold; *P* = 0.043) were observed in diabetic corneas compared with normal corneas. An approximately 1.5-fold reduction in *Abcg2* was also observed, although this was not statistically significant. *18S ribosomal RNA* expression showed no significant difference between the normal group and group with diabetes (*P* = 0.254). To further compare protein expression of corneal stem/progenitor markers, we performed immunofluorescence microscopic studies in normal and diabetic corneas. The expression levels of Hes1 and Keratin19 was examined in normal corneal limbal cells (Figures 3(a) and 3(c)). The expression levels of both these stem cell markers decreased in the diabetic corneas (Figures 3(b) and 3(d)).

### 3.3. Effects of rhIGF-1 Administration on Corneal Innervation and Corneal Progenitor/Stem Cells

**Figure 4:**
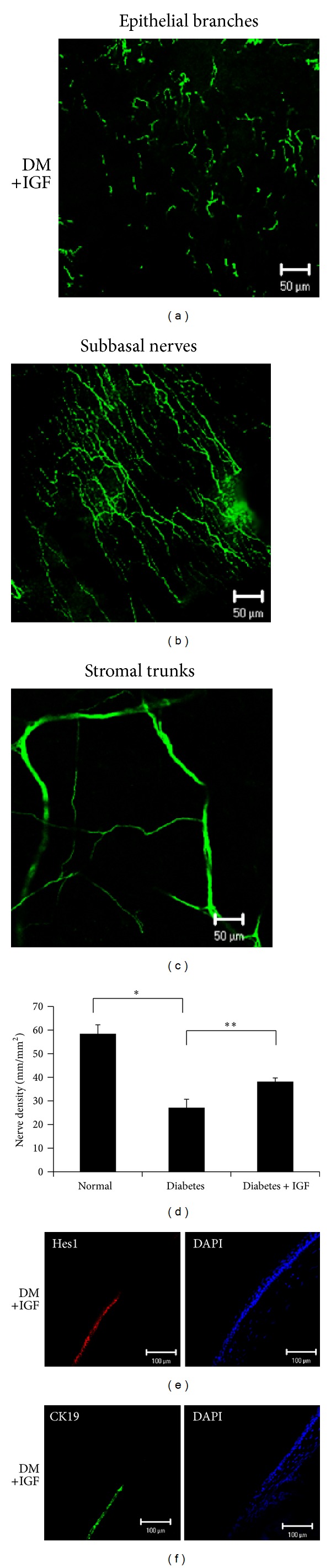
Effect of diabetes and treatment with recombinant human insulin-like growth factor-I (rhIGF-1) on corneal innervation and corneal progenitor/stem cells. Representative histological photomicrographs of corneal nerves that were immunostained with *β*-III-tubulin showing epithelial nerve (a), the subbasal nerve plexus (b), and stromal nerve trunks. (c) The rhIGF-I administration significantly accelerated recovery of the corneal subbasal nerve and epithelial branches. (d) Histogram of the quantification of the corneal subbasal nerve density. The subbasal nerve plexuses of the diabetic eyes completely diminished compared with that in the normal eyes, whereas those of rhIGF-1-treated diabetic corneas recovered. Statistical comparison of data between groups was performed using the nonparametric Mann-Whitney *U* test. The bars represent the mean ± SEM (*P < 0.00001, **P < 0.01). Representative immunostained micrographs of cross-sections of diabetic and rhIGF-1-treated corneas showing the expression of progenitor/stem cell markers. The decrease in Hes1 (e) and Keratin19 (f) staining was prevented in rhIGF-1-treated diabetic mice compared with diabetic mice (Figures 3(b) and 3(d)).

Figure 4 provides representative illustrations of the nerve fibers innervating the corneal epithelium (a) and the subbasal layer (b) in rhIGF-1-treated diabetic mice. In rhIGF-1-treated diabetic mice, corneal innervations significantly increased. We compared the corneal subbasal nerve density between the normal and diabetic corneas (Figure 4(d)). The corneal subbasal nerve plexus density of normal corneas was 58.29 ± 3.94 mm/mm^2^, whereas that of diabetic corneas was 27.01 ± 3.70 mm/mm^2^ (*P* < 0.00001). The subbasal nerve density in rhIGF-1-treated diabetic mice significantly increased (38.06 ± 1.67 mm/mm^2^) compared with that in the diabetic mice (*P* < 0.01). There was no significant difference in the corneal stromal nerve trunk densities among the normal, diabetic mice, and rhIGF-1-treated diabetic mice (data not shown). Moreover, the decrease in Hes1 and Keratin19 staining was suppressed in the rhIGF-treated diabetic cornea (Figures 4(e) and 4(f)) compared with that in diabetic corneas (Figures 3(b) and 3(d)).

## 4. Discussion

In this study, we used db/db model mice and observed that the impaired corneal stem/progenitor cells that were observed in the type 2 diabetes models could have involved a loss of corneal nerves. Corneal nerves, in addition to their well-known sensory functions, help maintain the integrity of the ocular surface by releasing epitheliotrophic substances that promote corneal surface health [31]. In particular, the subbasal corneal nerve plexus contains the highest number of nerve branches in the cornea, and these branches directly extend into the corneal epithelium [32–34]. Prior studies have shown a reduction of the subbasal plexus in subjects with diabetes compared with normal subjects [35]. We also observed a decreased density of the corneal subbasal nerve plexus and corneal epithelial branches in leptin receptor mutant mice, which are an accepted animal model of type 2 diabetes. In this model, higher tortuosity was observed in the corneal subbasal nerve than in the normal nerve.

Diabetic corneal ulcerations remain an unresolved clinical condition. Diabetic patients suffer severe peripheral neuropathy, which is associated with the development of persistent corneal epithelial defects [36–38]. Although various nerve-secreted factors and peptides, such as NGF, substance P, and brain-derived neurotrophic factor, are important for epithelial regeneration and wound healing [39–44], corneal stem/progenitor cells are also closely related to corneal wound healing. However, Seitz et al. have shown that the underlying leptin deficiency in db/dbmice further amplifies these impairments in skin repair [45]. To characterize this, we studied the expression of putative corneal progenitor/stem cell markers and found a 60% reduction in p75, Keratin15, and Hes1 expression in diabetic corneas. In our study, not only were epithelial abnormalities and decreased innervation present in diabetic corneas but also the corneal epithelial progenitor/stem cells were altered and/or impaired. The high tear glucose concentration and various complications of diabetes make it difficult to fully dissociate the contribution of nerve depletion versus other pathological aspects of the disease with the stem cell reduction. However, given that the corneal limbus, where the corneal progenitor/stem cells reside, is densely innervated, delayed corneal epithelial wound healing may result, at least in part, due to impaired corneal progenitor/stem cells in diabetic corneas. Furthermore, these results supported those of our previous studies that the number of corneal stem/progenitor cells significantly decrease after deprivation of the sensory nerves [21].

With regard to physiological effects of diabetes, there are several studies that indicate a potential role for IGF, glucagon-like peptide-1 (GLP-1), and dipeptidyl peptidase inhibitors in neuroprotection. Our results demonstrated that the rhIGF-I-treated diabetes group exhibited a significantly accelerated recovery of the corneal subbasal nerve and epithelial branches. The corneal subbasal nerve tortuosity remained in the rhIGF-1-treated diabetic mice, although we observed an increase in the density of the subbasal nerves. With topical administration of rhIGF-1 in diabetic corneas, alterations in the expression patterns of the corneal stem/progenitor proteins became consistent with healing. The normal corneal epithelium is a stratified layer of cells connected by tight junctions that provide a barrier against compounds larger than 1 nm. The pharmacological action was considered to be poorly effective because the high molecular weight (e.g., rhIGF-1) made penetration of the corneal epithelium difficult. The ciliary nerves of the trigeminal nerve entering the sclera are anatomically located close to the optic nerve at the posterior globe. In previous reports, the sclera permeability was significantly higher than the respective permeability across the cornea and conjunctiva for macromolecular substances [46]. It was difficult to inject the precise volume and maintain the drug so that the mouse cornea could exhibit minimum thickness at the limbus. Furthermore, we aimed to inhibit the fluctuating or low concentration of the drug by tear fluid. Although there are no studies on the effectiveness of retrobulbar IGF-1 injections, we used a periocular approach for retrobulbar injection in order to avoid any delivery loss of the medicine because of frequent blink and to achieve high infiltration and drug retention in the periocular tissue.

Recently, Chen reported that treatment with rhIGF-I can promote hematopoietic stem cell/progenitor survival, as shown by the increased frequency of Sca-1+ cells in bone marrow harvested after subcutaneous injection with rhIGF-I following total body irradiation [47]. The direct neuronal (intrathecal) delivery of IGF-I improved and reversed the slowing of motor and sensory conduction velocity in rats with diabetes. In addition, insulin and IGF-I prevented atrophy in myelinated sensory axons in the sural nerve [48]. As they also mentioned, it is thought that the direct delivery of IGF-I to neurons may correct diabetes-associated abnormalities by restoring suitable mitochondrial function. Moreover, it has been demonstrated that rhIGF-I decreases apoptosis and promotes cell survival in a PI3-kinase-dependent manner [49]. IGF-I activates the serine/threonine kinase Akt through PI3-kinase. This activation phosphorylates and inhibits caspase-3, resulting in decreased apoptosis. Therefore, rhIGF-1 treatment could protect corneal stem/progenitor cells from damage. Taken together, our data suggested that the preservation of corneal stem/progenitor cells could be a consequence of regenerative changes in the corneal nerves in type 2 diabetes. With regard to the healing of corneal nerve and stem/progenitor cells, the topical administration of IGF-1 may become applicable in severe situations caused by persistent corneal ulcers in patients with type 2 diabetes.

## 5. Conclusion

In conclusion, our results suggest that the number of corneal progenitor/stem cells significantly reduce in an animal model of type 2 diabetes. We provided novel evidence for the critical role of innervation in maintaining corneal epithelial cells and/or the stem cell niche in diabetes. Furthermore, our studies showed that rhIGF-1 prevented and/or decreased changes in the corneal progenitor cells and nerves that were associated with type 2 diabetes. These findings were also innovative results, wherein alterations in the corneal nerve and stem/progenitor cells were consistent with healing in IGF-1-treated diabetes. It is provocative to guess that the functional relationship between the nerve and stem cells that was described in this paper could apply to other epithelial/nonepithelial stem cells in the body. Thus, these findings contributed to our understanding of the factors that are relevant to maintaining the progenitor/stem cell niche in diabetes.
